# Effects of Equine-Assisted Therapies or Horse-Riding Simulators on Chronic Pain: A Systematic Review and Meta-Analysis

**DOI:** 10.3390/medicina56090444

**Published:** 2020-08-31

**Authors:** Daniel Collado-Mateo, Ana Myriam Lavín-Pérez, Juan Pedro Fuentes García, Miguel Ángel García-Gordillo, Santos Villafaina

**Affiliations:** 1 Centre for Sport Studies, Rey Juan Carlos University, Fuenlabrada, 28943 Madrid, Spain; daniel.collado@urjc.es; 2 Physical Activity and Quality of Life Research Group (AFYCAV), Faculty of Sport Science, University of Extremadura, Cáceres, 10003 Extremadura, Spain; jpfuent@unex.es (J.P.F.G.); svillafaina@unex.es (S.V.); 3 Facultad de Administración y Negocios, Universidad Autónoma de Chile, Talca 3467987, Chile; miguelgarciagordillo@gmail.com

**Keywords:** pain, hippotherapy, horse-riding, simulator, equine-assisted therapy, horseback riding

## Abstract

*Background and objectives:* Chronic pain is a complex global public health problem that affects the health status, quality of life, activities of daily living, and different work-related variables. Riding a horse may lead to some benefits in chronic pain patients through the improvement of postural control and other biopsychosocial processes. Therefore, this systematic review and meta-analysis aimed to evaluate the effects of horse riding (with real or simulated horses) on chronic pain. *Materials and methods:* A systematic literature search was carried out in accordance with PRISMA guidelines in Web of Science (WOS) and PubMed (Medline) electronic databases. Eleven articles (seven randomized controlled trials) were selected to be included in the review. Due to some risk of bias concerns, two meta-analyses (using postintervention or change-from-baseline measures) were conducted utilizing Review Manager Software (RevMan 5.3). *Results:* Horse-riding simulators significantly reduced the pain levels of patients with low back pain (*p* = 0.03, with a SMD of −1.14 and a 95% CI from −2.16 to −0.11) using change-from-baseline measures. However, the *p*-value in the meta-analysis with the postintervention measures was 0.06. Regarding interventions with real horses, it was not possible to conduct a meta-analysis due to the low number of studies. *Conclusion:* Horse riding could be a useful exercise to reduce pain, but more studies are needed to make evidence-based recommendations and to compare the effects of horse-riding with real and simulated horses.

## 1. Introduction

Chronic pain is a global public health problem that involves a large burden, especially for primary care practitioners and hospital emergency departments [1,2]. In 2012, about 126 million adults from the United States (US) (≈55%) suffered from some pain in the last three months, while more than 25 million (≈11%) adults from the US [2] and around 19% of Europeans [3] had any kind of chronic pain. It was also observed that pain levels are positively associated with worse health status, higher risk of depression, more disabilities, higher risk of losing a job, and more need for healthcare services [4,5]. Chronic musculoskeletal pain is the most prevalent among chronic pain conditions [3], and the burden may be underestimated [6].

Chronic musculoskeletal pain can be classified as “primary” when pain is a condition itself, or “secondary”, when pain is a symptom of other diseases [7]. However, scientific evidence suggests that chronic pain should be treated as a complex biopsychosocial disease and not just a symptom [8]. Thus, there is a need for treatments not only focused on the reduction of pain but also able to improve other variables such as quality of life or the ability to perform activities of daily living. The recommendations for the treatment of chronic pain include an interdisciplinary, integrated, multimodal, and evidence-based approach involving pharmacological and nonpharmacological treatments [9].

Equine-assisted therapies (EAT) have emerged as a potential therapeutic alternative for different populations, such as attention deficit [10], cerebral palsy [11], autism [12], or older adults [13]. Among the equine-assisted therapies, hippotherapy uses the movement induced by the horse to provide benefits in the rider. The rider’s pelvis motion while riding is similar to the observed while walking, thus riding a horse, generates bilateral, continuous and symmetrical movement patterns that stimulate voluntary and involuntary muscular activity and helps the maintenance of adequate posture and balance [14]. This is especially useful as a treatment for neurological disorders such as cerebral palsy [15]. In the case of chronic back pain, riding a horse has shown to have potentially beneficial effects on functionality and activity [16]. Furthermore, horseback riding strengthens musculoskeletal and neurological factors improving balance, increasing the trunk/head stability, and functional improvements [17,18,19,20], which may be relevant in low back pain (LBP) patients.

However, EAT has some limitations and disadvantages that could complicate the practice. First, EAT are usually not covered by health insurance. Furthermore, this activity implies high costs to care for and train the horses (which often leads to large costs for users), the distance to riding centers (which are often placed in remote areas) as well as other limitations such as the weather, the risk of allergic reactions, or other aspects such as fear or anxiety to ride a horse [21]. These problems make it so that EAT could sometimes be challenging to maintain the treatment for a long time and, consequently, the benefits could not be completely achieved. Horse riding simulators have emerged as an alternative to EAT, attempting to emulate the movement and appearance of a real horse in a controlled indoor environment without the previously commented limitations. However, the emotions elicited by real horses are entirely different [22], and it has been suggested that the benefits of horse riding simulators could be limited to physical fitness and muscular activity [13], so their potential in a biopsychosocial disease could be reduced to the physical aspect.

Given the promising benefits of EAT for LBP patients, the current systematic review and meta-analysis aimed to evaluate the effects of EAT on chronic pain. Furthermore, the effects of therapies based on horse riding simulators were also investigated due to the similarities in movements provided by EAT and the possible accessibility advantages.

## 2. Materials and Methods

This systematic review has been registered in the International Prospective Register of systematic reviews (PROSPERO) with the following identification number: CRD42020191088. Moreover, the current systematic review has followed the PRISMA (Preferred Reporting Items for Systematic reviews and Meta-Analyses) guidelines [23].

### 2.1. Data Sources and Searches

The following database resources were used to collect the systematic review articles: PubMed (MEDLINE) and Web of Science (including Current contents connect, Derwent innovations index, Korean journal database, Medline, Russian science citation index, SciELO citation index). “Hippotherapy” or “equine-assisted” or “horse-riding” or “horseback riding” and “pain” were the terms used in the search. Only articles written in English were searched. Duplicated articles were manually excluded. The process ended on 9 July 2020.

The articles were incorporated into the analysis when the following inclusion criteria were met:

1. The study was a controlled trial.
2. The target population had any kind of pain.
3. The intervention program was based on EAT or mechanical horse simulators.
4. The study included before and after the intervention measures.

**Figure 1:**
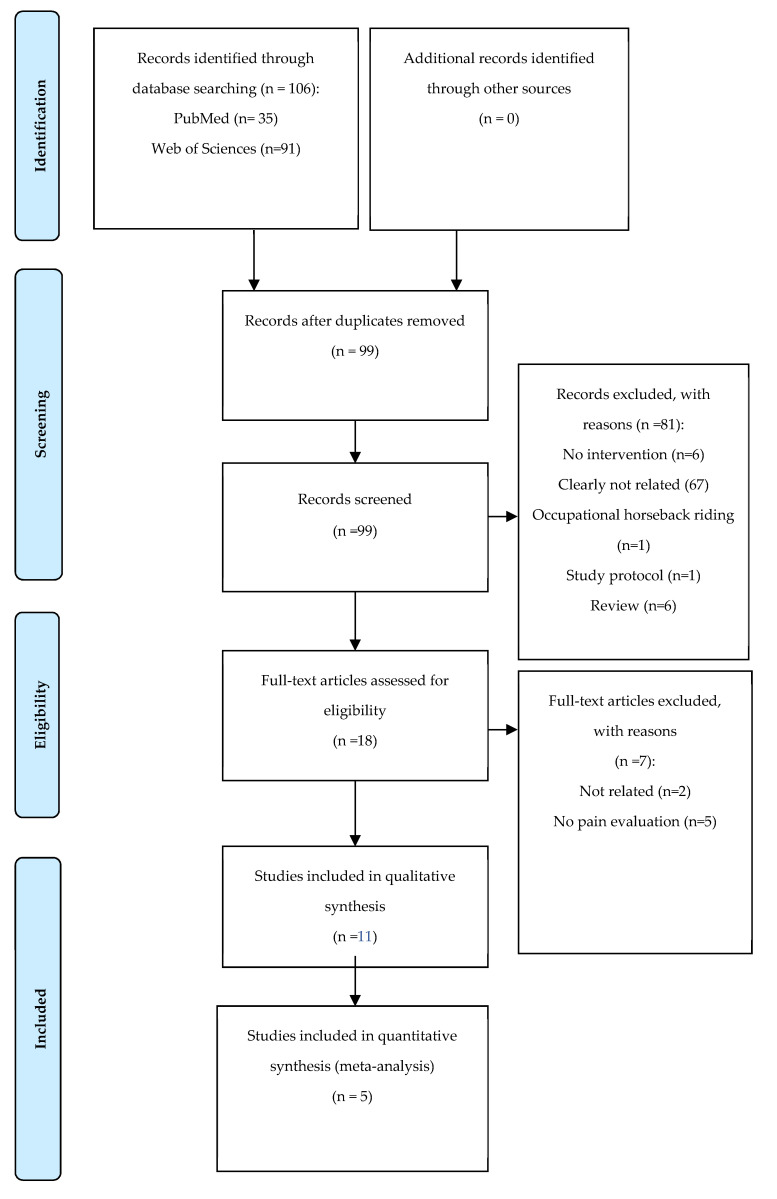
Study flow diagram.

Moreover, the next exclusion criteria were applied: (a) articles written in a language other than English or Spanish, and (b) studies not reporting changes or pre/post outcomes for pain. The selection of articles, shown in Figure 1, was done by one of the investigators implicated (DCM) and supervised by another author (AMLP).

Although the intention was to include only controlled trials, due to the low number of studies focused on EAT, uncontrolled studies were also included in the review but not in the meta-analysis and were reported separately to avoid confusion and misunderstandings.

### 2.2. Risk of Bias

The Evidence Project tool was used to assess the risk of bias [24]. The eight items instrument was designed to evaluate articles with quantitative data, including randomized and nonrandomized trials and control and uncontrol designs. Thus, the selected reliable tool evaluates the study design (three items), the participants’ representativeness (three items), and the equivalence of comparison groups (two items). Table 1 summarizes the risk of bias of randomized controlled trials and Table 2 shows the risk of bias of the rest of the studies.

### 2.3. Data Extraction

**Figure 2:**
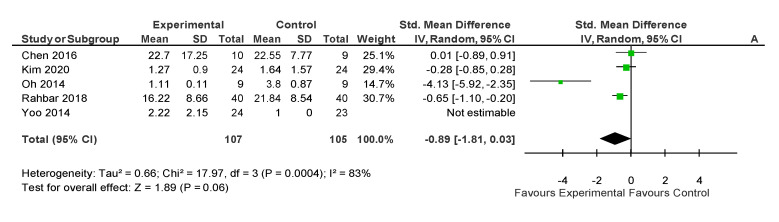
Results of after intervention pain outcomes.

The process to extract the information was performed by one of the authors (DCM) and checked afterward by another (AMLP). Data from the selected articles were extracted following PRISMA steps, collecting the participants, intervention, comparisons, results, and study design (PICOS) [23] information. Table 3 includes the type of study and participants baseline characteristics (sample size, age, pathology, pain duration, body mass index or weight, and disability level) while the description of the interventions (length, sessions duration, weekly frequency, setting, type of exercise, and exercise description) are presented in Table 4. For pain outcomes, the articles reported the Visual Analogue Scale (VAS) and the Numeric Pain Rating Scale (NPRS). Figure 2 shows the results from simulator riding intervention, whereas Table 5 shows the effects of EAT with real horse interventions. In addition, Table 6 shows the design, participants, intervention, and results of those articles that did not include a control group.

### 2.4. Outcome Measures

Between-group improvements were only found in the White-Lewis, Johnson, Ye and Russell [25] study when comparing the back pain level to the standard care or an educational program (Table 5). The rest of the significant differences observed after EAT interventions were always within-group differences [25,26].

### 2.5. Statistical Analysis

Although the use of after-intervention measures is recommended in meta-analyses [27], there were some differences at baseline in the included articles that could affect the results. Thus, both after intervention and change-from-baseline measures were used in the meta-analysis to check the results and reduce the influence of those differences [28]. Pain score means and standard deviations from the mechanical horse simulator group were contrasted with the control group outcomes. The Review Manager Software (RevMan 5.3) [29] was used to perform the analyses, selecting the inverse variance and random effects methods due to the heterogeneity of the results [30]. Standardized mean difference (SMDs) was utilized since different scales were applied to evaluate pain (VAS and NPRS). The Cochrane Handbook was used to interpret the SMDs outcomes, defining as small effects scores < 0.4, moderate effects from 0.4 to 0.7, and large effects > 0.7 [27]. Besides, the results obtained were represented with a confidence interval (CI) of 95%, and the heterogeneity was calculated by the I^2^ statistic model, and, for the overall effect, Z-test.

## 3. Results

### 3.1. Study Selection

The flow for search and selection of articles is depicted in Figure 1. By filtering the articles not written in English, a total of 99 potentially eligible articles were identified after removing the duplicated ones. Of these, 92 articles were excluded for different reasons. Most of the articles were excluded after reading the title and abstract because they were not related to the aim of this systematic review. Other articles required a full-text screening to ensure that they fulfill all the inclusion and exclusion criteria. Finally, eleven articles (seven controlled trials and four uncontrolled trials) were included in the systematic review, with five of them evaluating the effects of horse-riding simulators (five controlled trials) and six focused on EAT (two controlled trials and four uncontrolled trials). Thus, only the five articles that aimed to evaluate the effects of simulators were included in the meta-analysis.

### 3.2. Risk of Bias

Table 1 showed the risk of bias assessment of the included controlled studies. All of them fulfilled all items for study design and participant representativeness with the exception of item 5 “random selection of participants for assessment”. Regarding the equivalence of comparison group, there were some potential concerns: (1) in the study by White-Lewis, Johnson, Ye and Russell [25], one group was fully comprised by women and, in the other, 40% were men; (2) in the study by Kim, et al. [31], the mean duration of the pain symptoms in the two groups was 58.22 and 101.55, which might have affected the results; in the study by Vermöhlen, Schiller, Schickendantz, Drache, Hussack, Gerber-Grote and Pöhlau [26] and the study by Yoo, et al. [32], the baseline pain level was different between the included groups. In this last study, the control group showed a back pain of 1.50 while for the EAT group back pain was 4.37 at baseline. After the intervention, all participants from the control group had a score of 1.00, with a SD of 0.00.

Table 2 summarizes the scores of the included uncontrolled studies. The scores were low and the items 7 and 8 were not applicable due to the lack of a control group.

### 3.3. Study Characteristics

Table 3 shows the type of study and participants’ baseline characteristics (sample size, age, pathology, pain duration, body mass index or weight, and disability level) of the articles that included a control group. All those studies were randomized controlled trials. The two studies which aimed to evaluate the effects of EAT involved 87 adults with arthritis or multiple sclerosis. On the other hand, the five studies using horse riding simulators involved 231 patients suffering from LBP. Participants were older in the studies with real horses (mean or median age higher than 50) compared to the studies using simulators, aged lower than 30 in four of the five included studies [31,32,33,34] and ≈46 years in the remaining one [35].

### 3.4. Interventions and Comparison Groups

Table 4 shows the description of the interventions carried out in the randomized controlled trials. The interventions for the two studies with real horses were based on EAT [26], including riding, different tasks on the horse, and the grooming and taking care of the horse [25]. Thus, this last study included not only riding but also some activities to create a bond between the horse and the rider. The duration of this study was six weeks, while the study by Vermöhlen, Schiller, Schickendantz, Drache, Hussack, Gerber-Grote and Pöhlau [26] lasted 12 weeks. On the other hand, the studies using horse riding simulators had a duration of four to eight weeks. Another study [35] did not report the number of weeks but the number of sessions. The frequency went from twice a week [31] to five times a week [33].

**Figure 3:**
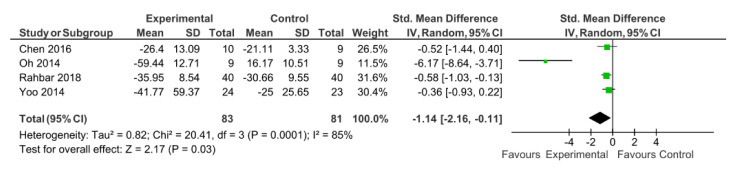
Results of change-from-baseline pain outcomes.

Comparison groups were different in the included studies. For those with real horses, control groups received education about exercise and arthritis [25] or continued their previous therapy [26]. Regarding studies using simulators, two studies [32,33] had an inactive control group who continued their usual care, and three studies with some kind of physical therapy [31,34,35]. Regarding the interventions based on horse simulators, two meta-analyses were conducted. In this regard, using the pain levels after the intervention, five articles were potentially eligible to be included. However, the study by Yoo, Kim, Lee, Jin, Hong, Choi, Kim and Jee [32] was excluded because the SD was 0.00 after the intervention. Results from the meta-analysis showed a *p*-value = 0.06, with a SMD of −0.89 (95% CI from −1.81 to 0.03) with large heterogeneity (I^2^ = 83%) (Figure 2). However, although it was a randomized controlled trial, in the study by Yoo, Kim, Lee, Jin, Hong, Choi, Kim and Jee [32], the baseline pain level was different between the included groups. Thus, the utilization of postintervention measures could be inappropriate. Therefore, a second meta-analysis was conducted using change measures. This meta-analysis showed a significant reduction of pain (*p* = 0.03) after an intervention based on a horse-riding simulator. The SMD was −1.14 (95% CI from −2.16 to −0.11) with large heterogeneity (85%) (Figure 3).

Apart from the randomized controlled trials, Table 6 summarizes the main characteristics of those articles that did not include a control group. Two case studies [36,37], one single-subject experimental design (reporting results patient by patient) [38] and one action research [16] were included. Overall, 41 subjects participated in these studies and the authors reported promising results, with pain reduction after EAT interventions.

## 4. Discussion

This systematic review and meta-analysis aimed to evaluate the effects of any kind of horse-riding activity (with real horses or simulators) on pain. The main finding was that horse-riding simulators are a promising tool to reduce pain levels in people suffering from LBP. However, although the included studies were randomized controlled trials, the interpretation of results must be done with extreme caution due to the large heterogeneity, the low number of studies, and the potential risk of bias. The results using postintervention and change measures showed the same tendency with an SMD classified as large (i.e., >0.7) but, only when the post-pre differences are used, the SMD reached the statistical significance (0.03 vs. 0.06 when after intervention measures are used). Regarding EAT studies, between-groups differences were only observed in one of the selected articles [25], while the rest of the included studies reported promising results based on within-group or within-subject analyses. However, although promising, the current evidence for the benefits of EAT on pain is still low and some contradictory findings can be observed.

Horse-riding simulators have emerged as an alternative for real horse riding since they could provide a comparable pattern of stimuli which can lead to specific postural responses [39]. It must be noted that this type of therapy could have some objectives advantages [21], involving lower costs due to the maintenance of the machine is cheaper than the costs associated with caring and training the horses. Furthermore, the facilities needed to carry out the sessions with real horses must be much larger, which makes it so that most hippotherapy facilities are placed out of the urban centers. Other aspects, such as the weather, the risk of allergic reactions, or the potential fear or anxiety to ride a horse must also be considered. However, horse riding simulators are limited to the imitation of the horse movement. In this regard, the emotional response of riding a real or a simulated horse is different [22]; the natural temperature of the horse (1° to 5° higher than human’s body temperature) may have some added benefits, such as reduced muscle spasticity and hypertonicity and the outdoor environment could also motivate and increase the pleasure [40,41].

Considering chronic pain as a biopsychosocial condition, therapies based on real horses were expected to lead to larger benefits than horse-riding simulators. This is due to the known positive effects of EAT on different psychological variables such as self-esteem, self-regulatory ability, empowerment, or competency, as well as the enhanced emotional wellbeing and the social benefits [41,42], which are associated with the bond between rider and horse. In this regard, Aldridge, Morgan and Lewis [37] pointed out that the motivation in EAT activities was very high. This can be explained by the characteristics of the intervention conducted in that study, which included not only riding the horse but also brushing or grooming so the participant can create a bond with the horse. Thus, this point could make the difference between EAT and horse-riding simulators. However, studies comparing the effects of real and simulated horses were not found and are certainly needed.

This systematic review and meta-analysis include participants suffering from different sources of pain. In this regard, studies with real horses involved patients with arthritis [25], multiple sclerosis [26,38], or back or neck pain [16,36,37], whereas patients in studies with simulated horses had LBP. However, in the meta-analyses all the articles included patients with LBP. In this regard, LBP is the most common among all types of chronic musculoskeletal pain conditions [43] and pain could be related to poor postural control, characterized by altered activation of the trunk muscles, reduced trunk movement, poor proprioceptive perception, stiffening, and postural instability [44,45,46,47]. Previous studies using both real or simulated horses have reported significant improvements in the postural control [48], muscle size of the transverse abdominal and lumbar multifidus [49], or isokinetic strength of the trunk and hip muscles [32,33]. These reasons could be behind the improvements of pain that we observed in the meta-analysis. However, further studies are needed to corroborate these potential relationships.

Some limitations could affect the present systematic review and meta-analysis. First, only eleven articles (seven randomized controlled trials) were included, and the sources of pain (LBP, neck pain, arthritis, and multiple sclerosis) and interventions (real and simulated horses) were different. Second, it was not possible to compare the two types of interventions (EAT and simulators), and the available data do not allow the extraction of conclusions about the different benefits of EAT and simulators in patients suffering from pain. Thus, further studies which compare the effects of real and simulated horses’ interventions are encouraged. Another limitation is the risk of bias of the included studies, which makes that the interpretation of results must be taken with caution. Although all included studies in the meta-analysis were randomized controlled trials, there were some differences at baseline that could have affected the results.

Considering these limitations, further studies which compare the effects of real and simulated horses’ interventions are encouraged. Furthermore, these interventions should include larger and more homogeneous samples, as well as randomized controlled trial designs. Outcomes which include all psychological, physiological, and psychosocial outcomes are encouraged to clarify the different effects of EAT and simulated horse interventions.

## 5. Conclusions

This systematic review and meta-analysis is, to the best of our knowledge, the first to evaluate the effects of horse riding on pain levels. Promising results were achieved, with a large SMD, and the potential mechanisms are discussed. More studies are needed to compare the effects of EAT and horse-riding simulators.

## Figures and Tables

**Table 1:** Risk of bias assessment using the Evidence Project tool.

Study	Study Design			Participant Representativeness			Equivalence of Comparison Groups		Total Score
	Item 1	Item 2	Item 3	Item 4	Item 5	Item 6	Item 7	Item 8	
**Real horse studies**									
White-Lewis et al. (2019)	Yes	Yes	Yes	Yes	No	Yes	No	Yes	6/8
Vermöhlen et al. (2018)	Yes	Yes	Yes	Yes	No	Yes	Yes	No	6/8
**Horse riding simulator studies**									
Rahbar et al. (2018)	Yes	Yes	Yes	Yes	No	Yes	Yes	Yes	7/8
Kim et al. (2020)	Yes	Yes	Yes	Yes	No	yes	Yes	Yes	7/8
Chen et al. (2016)	Yes	Yes	Yes	Yes	No	Yes	No	Yes	6/8
Yoo et al. (2014)	Yes	Yes	Yes	Yes	No	Yes	Yes	No	6/8
Oh et al. (2014)	Yes	Yes	Yes	Yes	No	Yes	Yes	Yes	7/8

Item 1: Cohort; Item 2: Control or comparison group; Item 3: Pre/post intervention data; Item 4: Random assignment of participants to intervention; Item 5: Random selection of participants for assessment; Item 6: Follow-up rate of 80% or more; Item 7: Comparison groups equivalent on sociodemographics; Item 8: Comparison groups equivalent at baseline on disclosure.

**Table 2:** Risk of bias assessment of the uncontrolled studies using the Evidence Project tool.

Study	Study Design			Participant Representativeness			Equivalence of Comparison Groups		Total Score
	Item 1	Item 2	Item 3	Item 4	Item 5	Item 6	Item 7	Item 8	
**Real horse studies**									
Wehofer et al. (2013)	Yes	No	Yes	No	No	Yes	N/A	N/A	3/6
Hammer et al. (2005)	Yes	No	Yes	No	No	Yes	N/A	N/A	3/6
Aldridge Jr. et al. (2016)	Yes	No	No	No	No	Yes	N/A	N/A	2/6
Hakanson et al. (2009)	Yes	No	No	No	No	Yes	N/A	N/A	2/6

Item 1: Cohort; Item 2: Control or comparison group; Item 3: Pre/post intervention data; Item 4: Random assignment of participants to intervention; Item 5: Random selection of participants for assessment; Item 6: Follow-up rate of 80% or more; Item 7: Comparison groups equivalent on sociodemographics; Item 8: Comparison groups equivalent at baseline on disclosure. N/A: Not applicable.

**Table 3:** Type of study and participants’ baseline characteristics of the selected articles.

Study	Design	Group	Sample Size (% of Females)	Age (SD)	Pathology	Pain Duration	BMI/Weight	Disability Level
**Real horse studies**								
White-Lewis et al. (2019)	RCT	Inactive control group	n = 10 (100%)	65.80 (7.42)	Arthritis	NR	NR	NR
		Equine-assisted therapy group	n = 10 (60%)	61.90 (6.05)		NR	NR	NR
Vermöhlen et al. (2018)	RCT	Inactive control group	n = 37 (73%)	51 (47–56) *	Multiple sclerosis	17.6 (11–27) years *	70.6 (9.9) kg	EDSS < 5: 303% EDSS ≥ 5: 70%
		EAT group	n = 30 (90%)	50 (45–53) *		16.5 (11–20) years *	67 (10.3) kg	EDSS < 5: 33% EDSS ≥ 5: 67%
**Horse riding simulator studies**								
Kim et al. (2020)	RCT	Active control group	n = 24 (57.7%)	28.76 (9.05)	Low back pain	101.55 (97.12) months	23.50 (5.58) kg/m^2^	KODI: 21.77 (7.11) KRMD: 5.11 (2.74)
		Simulator group	n = 24 (31.8%)	26 (3.82)		58.22 (37.37) months	23.96 (5.76) kg/m^2^	KODI: 20.24 (7.69) KRMD: 5.11 (2.74)
Rahbar et al. (2018)	RCT	Inactive control group	n= 40 (27.5%)	46.22 (7.83)	Low back pain	7.22 (1.79) months	26.89 (0.50) kg/m^2^	KRMD: 15.32 (0.24)
		Simulator group	n = 40 (32.5%)	46.25 (7.97)		7.05 (1.74) months	26.96 (0.55) kg/m^2^	KRMD: 15.50 (0.26)
Chen et al. (2016)	RCT	Active control group	n = 9	19–30	Nonspecific chronic low back pain	<3 months	NR	KODI: 10.55 (4.06)
		Simulator group	n = 10				NR	KODI: 9.60 (3.53)
Yoo et al. (2014)	RCT	Inactive control group	n = 23 (0%)	20.70 (1.45)	Chronic low back pain	8.35 (2.62) months	Weight 65.80 (7.38) kg.	
		Simulator group	n = 24 (0%)	20.44 (1.33)		9.41 (3.64) months	Weight 64.69 (9.96) kg.	NR
Oh et al. (2014)	RCT	Inactive control group	n = 9 (0%)	20.70 (0.37)	Chronic low back pain	6.38 (2.14) months	Weight 65.80 (2.40) kg.	
		Simulator group 1	n = 10 (0%)	20.56 (0.69)		6.21 (2.11) months	Weight 69.92 (4.87) kg.	NR
		Simulator group 2	n = 9 (0%)	20.33 (0.52)		7.57 (1.6) months	Weight 60.92 (2.56) kg.	
		Simulator group 3	n = 9 (0%)	20.44 (0.27)		6.75 (2.01) months	Weight 64.41 (3.36) kg.	

KODI: Korean Oswestry disability index; KMD: Korean Roland Morris disability EDSS: Expanded Disability Status Scale; NR: not reported; * Median and interquartile range.

**Table 4:** Description of the interventions.

Study	Group	Length (Weeks)	Sessions Duration (min)	Frequency (Times/Week)	Setting	Type of Exercise	Exercise Description
**Real horse studies**							
White-Lewis et al. (2019)	Control group	6	60	1	Nursing school	Education sessions of exercise	Evidence-based exercise education for adults and older adults with arthritis
	EAT group	6	60	1	Certified riding stables with the supervision o 2 staff members	EAT	Warm-up: stretching exercises such as knee lifts, ankle rolls, and hand to opposite knee touches. Riding: either steps or a ramp at the level of the horse’s back allowing participants to sit backwards and swing their right leg over the saddle and horse. Riding tasks (30 min): of increasing difficulty. Grooming and giving treats to the horse after riding
Vermöhlen et al. (2018)	Control group	12				Continue their previous therapy	
	EAT group	12	30	1	Five sites in Germany	EAT	Hippotherapy (as defined by the regulations of the Deutsches Kuratorium für Therapeutisches Reiten
**Horse riding simulator studies**							
Kim et al. (2020)	Control group	8	46	2	NR	Stabilization exercise with suspension	Warm up (5 min): stretching Workout (30 min): Supine pelvic lift, birding exercise, Side-lying hip abduction Rest time (6 min) Cool- down (5 min): stretching
	Simulator group	8	46 (exercise part)	2	NR	Horse riding simulation	Warm up (5 min): stretching Workout (30 min): Walking (80 m/min), slow trot (135 m/min), fast trot (159 m/min) Rest time (6 min). Cool- down (5 min): stretching
Rahbar et al. (2018)	Control group	15 sessions		NR	Physical therapy center	Physiotherapy	Physical modalities (surface heat, deep heat, and transcutaneous electrical nerve stimulation) + therapeutic exercise (lumbar and core stabilizing and strengthening, and lower back stretching
	Simulator group	30 sessions	Riding 15 min	NR	Physical therapy center	Physiotherapy + Mechanical horse simulator	Preparatory mode riding
Chen et al. (2016)	Control group	4		3	NR	Core stretching	6 movements repeated 5 times/set Each movement: 8 repetitions of 5 s
	Simulator group	4	30 min (15 min each modality)	3	NR	Horse riding simulation + core stretching	Riding: simulating riding a real horse through the visual information that appeared on the front screen by diving the virtual environment. Core stretching: 6 movements repeated 5 times/set. Each movement: 8 repetitions of 5 s
Yoo et al. (2014)	Control group	8				Usual care	
	Simulator group	8	From 10 to 40 min (increase 10/2 weeks)	3	NR	Horse riding simulation	Warm up (10 min): stretching Riding (from 10 to 40 min): Ordinary walking (0.16 km/min), Sitting trotting (0.52 km/min), Rising trotting (0.877 km/min), Cantering (1 km/min) Cool down (10 min): stretching
Oh et al. (2014)	Control group	8				Usual care	
	Simulator group 1	8	20	5	NR	Horse riding simulation	Warm up (5 min): stretching Riding: ordinary walking (5 min at 0.16 km/min), sitting trotting (5 min at 0.52 km/min), rising trotting (5 min at 0.877 km/min) Cool down (10 min): stretching
	Simulator group 2	8	30	5	NR	Horse riding simulation	Warm up (5 min): stretching Riding: ordinary walking (5 min at 0.16 km/min), sitting trotting (10 min at 0.52 km/min), rising trotting (10 min at 0.877 km/min) Cool down (10 min): stretching
	Simulator group 3	8	40	5	NR	Horse riding simulation	Warm up (5 min): stretching Riding [25]: ordinary walking (5 min at 0.16 km/min), sitting trotting (15 min at 0.52 km/min), rising trotting (15 min at 0.877 km/min) Cool down (10 min): stretching

NR: Not reported; EAT: equine assisted therapy.

**Table 5:** Effects from intervention with real horses.

Study	Group	Questionnaire Used	Baseline		After Intervention		Change		*p* Values
			Mean	SD	Mean	SD	Mean	SD	
White-Lewis et al. (2019)	Inactive control group	VAS 1–100 mm back	39.00	28.63	29.60	20.93	−9.4	NR	WG *p* = 1.00
		VAS 1–100 mm knee	43.90	25.74	37.60	27.30	−6.3	NR	WG *p* = 0.93
		VAS 1–100 mm hip	34.30	26.31	24.80	19.70	−9.5	NR	WG *p* = 0.12
		VAS1–100 mm shoulder	17.80	11.35	20.00	22.49	2.2	NR	WG *p* = 0.53
	EAT group	VAS 1–100 mm back	41.10	30.60	14.80	18.47	−26.3	NR	BG *p* = 0.021 * WG *p* = 0.006 *
		VAS 1–100 mm knee	46.10	30.59	24.40	26.51	−21.7	NR	BG *p* = 0.27 WG *p* = 0.06
		VAS 1–100 mm hip	43.90	37.07	24.80	19.70	−19.1	NR	BG *p* = 0.23 WG = *p* = 0.027 *
		VAS 1–100 mm shoulder	48.90	38.07	16.10	21.47	−32.8	NR	BG *p* = 0.45 WG *p* = 0.007 *
Vermöhlen et al. (2018)	Inactive control group	VAS 1–100	24.7	29.3	23.4	27	−1.3	28	
	EAT group	VAS 1–100	32.3	29.9	24.9	27.6	−7.4	16.8	BG *p* = 0.055

* *p*-value < 0.05. VAS: Visual Analogue Scale; WG: Within groups; BG: between groups; SD: standard deviation.

**Table 6:** Design, participants, intervention, and results of those articles that did not include a control group.

Study	Design	Participants	Intervention	Results
Wehofer et al. (2013)	Case study	A 76-year-old women	10 weeks of weekly, 45 min sessions. The sessions consisted of riding a horse, at different velocities, led by a therapist.	A reduction in back pain from 5/10 to 0–2/10
Hammer et al. (2005)	Single-subject experimental design (SSED), type A-B-A	11 subjects (9 women) aged 47.9 (8.4) with multiple sclerosis	Ten weekly therapeutic riding sessions lasting 30 min. The sessions consisted of different exercises including trunk rotation or balance components and riding without visual input.	Three subjects who initially reported pain reported some pain reduction related to the intervention.
Aldridge Jr et al. (2016)	Case report	A 34-year-old male military veteran with low back and neck pain	One-hour hippotherapy session involved retrieving and returning the horse from the pasture or stall; tacking and untacking the horse, brushing and grooming; mounting and dismounting; and riding the horse performing strengthening and stretching exercises; changing directions and speeds.	The subject reported decreased low back and neck pain following hippotherapy sessions.
Hakanson et al. (2009)	Action research (the researcher acted with the possibility of introducing changes during the study)	28 patients (19 women) with neck and/or back pain	The average length of the treatment was 3.5 months. The number of treatments varied from 2 to 32, ranging from 5 to 45 min. Goals were set in relation to the patient’s functional limits and current riding skills. The sessions involved riding a horse with focus on body awareness.	Four participants dropped out due to fear or pain increase. There were promising but contradictory findings, with some patients increasing their pain intensity and others experiencing an increment.
